# miR-122–based therapies select for three distinct resistance mechanisms based on alterations in RNA structure

**DOI:** 10.1073/pnas.2103671118

**Published:** 2021-08-12

**Authors:** Jasmin Chahal, Luca F. R. Gebert, Carolina Camargo, Ian J. MacRae, Selena M. Sagan

**Affiliations:** ^a^Department of Microbiology & Immunology, McGill University, Montréal, QC H3G 1Y6, Canada;; ^b^Department of Integrative Structural and Computational Biology, The Scripps Research Institute, La Jolla, CA 92037;; ^c^Department of Biochemistry, McGill University, Montréal, QC H3G 1Y6, Canada

**Keywords:** hepatitis C virus, miR-122, resistance-associated variants, riboswitch, internal ribosomal entry site

## Abstract

Hepatitis C virus (HCV) is a positive-sense RNA virus that interacts with a liver-specific microRNA called miR-122. miR-122 binds to two sites in the 5′ untranslated region of the viral genome and promotes HCV RNA accumulation. This interaction is important for viral RNA accumulation in cell culture, and miR-122 inhibitors have been shown to be effective at reducing viral titers in chronic HCV-infected patients. Herein, we analyzed resistance-associated variants that were isolated in cell culture or from patients who underwent miR-122 inhibitor–based therapy and discovered three distinct resistance mechanisms all based on changes to the structure of the viral RNA. Specifically, resistance-associated variants promoted riboswitch activity, genome stability, or positive-strand viral RNA synthesis, all in the absence of miR-122. Taken together, these findings provide insight into the mechanism(s) of miR-122–mediated viral RNA accumulation and provide mechanisms of antiviral resistance mediated by changes in RNA structure.

## Significance

MicroRNA (miRNA)–based drugs are quickly taking the clinic by storm. Herein, we analyzed resistance-associated variants (RAVs) to the first miRNA inhibitors to make it to the clinic, namely miR-122 inhibitors for chronic hepatitis C virus (HCV) infection. We uncovered three distinct resistance mechanisms based on unique alterations to the structure of the viral RNA. Specifically, RAVs altered the structure of the viral RNA in a manner that promotes riboswitch activity, genome stability, or positive-strand viral RNA synthesis. Our findings support recent models of miR-122–mediated HCV RNA accumulation and provide mechanism(s) of resistance to antiviral therapy. These early insights into the mechanism(s) of resistance to miRNA-based therapies may be of importance as more miRNA-targeted therapies enter into the clinic.

Hepatitis C virus (HCV) is a positive-sense RNA virus of the *Flaviviridae* family. The ∼9.6 kb HCV genomic RNA consists of a single open reading frame, which gives rise to the viral polyprotein that is processed into the 10 mature viral proteins flanked by highly structured 5′ and 3′ untranslated regions (UTRs) (1, 2). As a positive-sense RNA virus, the viral genome itself must serve as a template for the different stages of the viral life cycle, including viral translation, replication, and packaging (2). To this end, the viral 5′ and 3′ UTRs contain several *cis*-acting RNA elements that play important roles in directing the various stages of the viral life cycle (2–4). Specifically, the 5′ UTR contains the viral internal ribosomal entry site (IRES) made up of stem–loops (SL) II-IV, which is required for viral translation, while sequences and SL structures in both the 5′ (SLI-II) and 3′ UTRs (variable region, polyU/UC-tract, and 96-nt X-tail) are required for viral RNA replication (2, 5–8). Additionally, the 5′ terminus of the HCV genome interacts with the highly abundant, liver-specific human microRNA (miRNA), miR-122 (9–12).

**Fig. 1.**
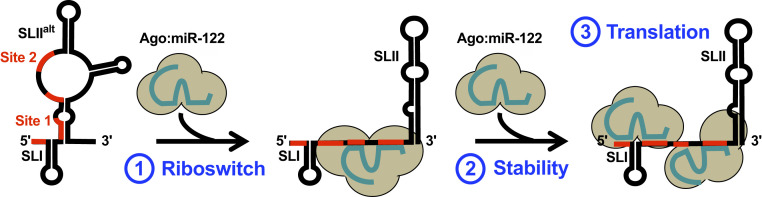
Model of miR-122 interactions with the HCV genome. The HCV genomic RNA is thought to enter the cell in an energetically stable conformation termed SLII^alt^. The recruitment of the first Ago:miR-122 molecule to the accessible (unpaired) site 2 serves as an RNA chaperone, akin to a bacterial riboswitch, which refolds the RNA into the functional SLII conformation and allows the viral IRES (SLII-IV) to form (*1*). Subsequent recruitment of a second Ago:miR-122 molecule to site 1 promotes genome stability by protecting the 5′ terminus from cellular pyrophosphatases and exoribonuclease-mediated decay (*2*). In order to accommodate the Ago:miR-122 complex at site 1, the Ago:miR-122 complex at site 2 releases its auxiliary interactions but is likely stabilized by interactions between the Ago protein and SLII of the HCV IRES (*3*). Collectively, these interactions promote HCV IRES-mediated translation. miR-122 seed and auxiliary binding sequences are indicated (red).

miR-122 is a highly expressed miRNA in the liver with ∼135,000 copies per hepatocyte (9, 13). While miRNAs typically interact with the 3′ UTRs of their target messenger RNAs (mRNAs) to dampen gene expression, miR-122 interacts to two sites in the 5′ UTR (site 1 and site 2) of the viral genome, and these interactions promote viral RNA accumulation (9, 10, 12). Several recent studies have led to a new model for miR-122:HCV RNA interactions that suggest that miR-122 plays at least three roles in the HCV life cycle (Fig. 1) (14–17). Firstly, the HCV 5′ UTR is thought to initially adopt an energetically favorable conformation (termed SLII^alt^), which results in the recruitment of an Ago:miR-122 complex to site 2 of the HCV genome. This results in an RNA chaperone-like switch in conformation, akin to a bacterial riboswitch (18), resulting in the formation of SLII and allowing the viral IRES (SLII-IV) to form (15–17). Secondly, this change in conformation allows the recruitment of Ago:miR-122 to site 1, which protects the 5′ terminus from pyrophosphatase activity and subsequent exoribonuclease-mediated decay (12, 14, 19–21). Finally, the Ago protein bound to site 2 makes direct contact with the viral IRES, promoting HCV IRES-mediated translation (16).

**Fig. 2.**
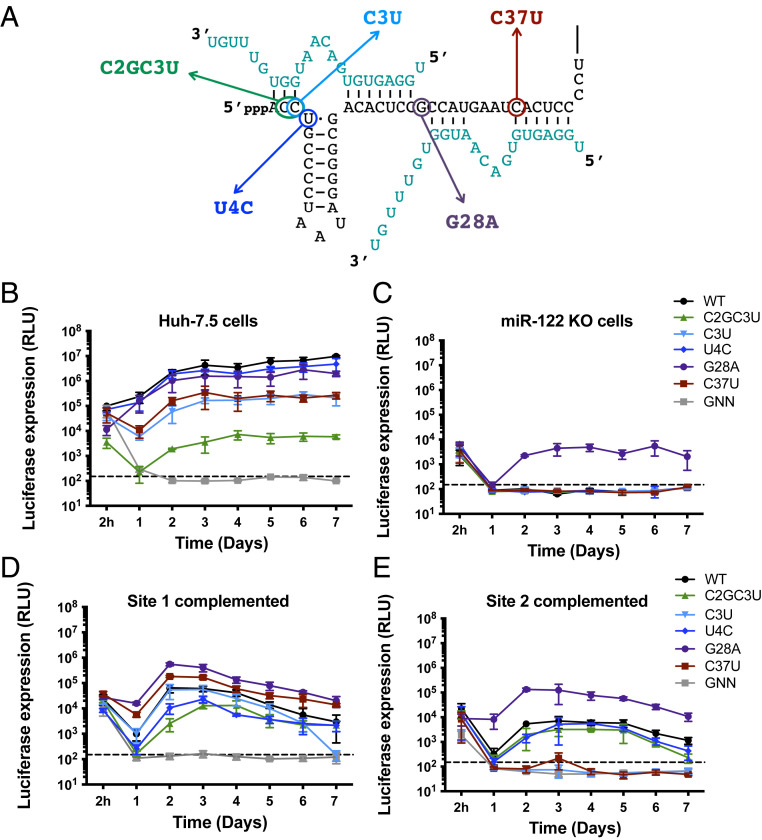
RAV accumulation in cell culture. (*A*) The positions of the RAVs on the 5′ UTR of the HCV RNA. Nucleotides 1 to 45 of the HCV genome (black) and the binding topology of the two miR-122 molecules (teal) are shown. Full-length RLuc HCV genomic reporter RNAs (WT and RAVs) were coelectroporated with a capped Firefly fuciferase (FLuc) mRNA into (*B*) Huh-7.5 or (*C*) miR-122 KO cells. Full-length RLuc HCV genomic reporter RNAs containing mutations at (*D*) site 1 (S1:p3A) or (*E*) site 2 (S2:p3A) were coelectroporated with a capped FLuc mRNA and compensatory miR-122p3U molecules into miR-122 KO cells. Luciferase activity was measured at the indicated time points post-electroporation. The limit of detection is indicated, and all data are representative of at least three independent replicates. Error bars represent the SD of the mean.

Due to the importance of miR-122 in the HCV life cycle, two miR-122 inhibitors (antisense oligonucleotides), the first miRNA-based drugs to enter clinical trials, have been developed and used to treat chronic HCV infection in the clinic (22, 23). Both Miravirsen (Santaris Pharma, a/s) and RG-101 (Regulus Therapeutics) miR-122 inhibitors have completed Phase II or Ib clinical trials, respectively, to investigate their clinical efficacy in chronic HCV infection (22, 23). Excitingly, both treatments led to dose-dependent and sustained reductions in viral loads; and, in the latter study, two patients achieved a sustained virological response (at least up to 76 wk posttreatment) after receiving a single dose of RG-101 (23). Neither treatment was associated with significant adverse events or long-term safety issues, suggesting that antisense targeting of miR-122 may be an effective treatment that could be used in future combination therapies. Interestingly, while no resistance was apparent during treatment, when viral RNA rebounded after the cessation of the inhibitor, several resistance-associated variants (RAVs) were identified in the 5′ UTR of the HCV genome (23). Specifically, C3U (genotype 1) was identified as a RAV in both the Miravirsen and RG-101 trials, while the C2GC3U (genotype 3/4) RAV was identified in the RG-101 trial alone, with both RAVs identified in multiple patients (22, 23). Additional RAVs were identified in cell culture, including studies performed with genotype 1 (A4C) and genotype 2 (U4C, G28A, and C37U), which were identified alone (A4C and G28A) or in combination with other RAVs (i.e., G28A+C37U, U4C+G28A+C37U) (24–26). As the cell culture–based studies identifying RAVs were performed with genotype 2 and the majority of the RAV nucleotide identities are present in genotype 2 (save for A4C, although U4C was deemed an equivalent RAV observed in this genotype), herein, we explored the mechanism of action of these collective RAVs (C2GC3U, C3U, U4C, G28A, and C37U) using genotype 2a (J6/JFH-1) reporter RNAs (Fig. 2*A*) (22–26). Previous work suggests that the G28A mutation is “riboswitched” and promotes the formation of the functional SLII structure even in the absence of miR-122 (16, 17). Similar to G28A, we hypothesized that the other RAVs also alter the structure of the viral RNA in a manner that negates the requirement for one or more miR-122 activities. Thus, we sought to provide insight into the mechanism(s) of action of the RAVs using RNA structure analysis and assays for viral RNA accumulation and decay. Our analyses suggest that each of the RAVs alter the structure of the viral RNA, and we identify three distinct resistance mechanisms based on unique changes in viral RNA structure.

## Results

### RAVs Do Not Have a Fitness Advantage in the Presence of miR-122 but Are Able to Accumulate under Conditions Where miR-122 Is Absent or Limiting.

Each of the HCV RAVs were isolated under conditions in which miR-122 was absent and/or limiting in cell culture or in miR-122 inhibitor–treated patients (22–26). Thus, we first sought to assess their ability to accumulate in cell culture in comparison with wild-type (WT) HCV RNA (Fig. 2 and *SI Appendix*, Fig. S1). First, we assessed viral RNA accumulation in miR-122 replete conditions in WT Huh-7.5 cells (Fig. 2*B*). In the presence of miR-122, WT HCV RNA accumulates to the greatest extent, while GNN (replication-defective viral RNA) is quickly decayed. WT HCV RNA accumulation was closely followed by U4C and G28A, with the C3U and C37U RAVs accumulating to a lesser extent, with ∼37.2- and 13.8-fold reductions in viral RNA accumulation at day 2 post-electroporation compared to WT (Fig. 2*B* and *SI Appendix*, Fig. S1*A*). The C2GC3U RAV accumulated to the least extent, with a 1,166.5-fold reduction in luciferase activity, although this was still significantly above background. Thus, under miR-122 replete conditions, WT and U4C have a fitness advantage when compared with the other RAVs.

Next, we asked whether we could detect viral RNA accumulation of any of the RAVs in the absence of miR-122 using miR-122 knockout (KO) Huh-7.5 cells (Fig. 2*C*). In this case, both WT and the majority of the RAVs quickly decayed, similar to GNN, with the exception of the G28A RAV (Fig. 2*C*). Interestingly, G28A was the only RAV able to accumulate in miR-122 KO cells, albeit at levels ∼1,000-fold lower than WT HCV RNA accumulation in Huh-7.5 cells (Fig. 2 *C* versus *B* and *SI Appendix*, Fig. S1 *A* and *B*). However, it is possible that the other RAVs are also able to accumulate but to levels that are below the level of detection in this assay. Nonetheless, these results suggest that only G28A has a distinct fitness advantage in viral RNA accumulation in the complete absence of miR-122.

As the RAVs identified in both cell culture and miR-122 inhibitor-treated patients were selected under conditions in which miR-122 was limiting, but not necessarily completely absent, we decided to investigate HCV RNA accumulation under conditions in which miR-122 was limiting (23–25). To do so, we introduced point mutations into the viral RNAs at either site 1 (C26A, Fig. 2*D*) or site 2 (C41A, Fig. 2*E*) and coelectroporated the viral RNAs with miR-122 molecules containing compensatory point mutations at position 3 (miR-122p3U) into miR-122 KO cells. Importantly, these specific seed site mutations were identified because they do not result in any predicted change to the conformation of the viral RNA (based on RNA structure prediction analyses), and previous studies suggest that mutations in the seed sequence are sufficient to abolish miR-122 interactions (12, 16). When miR-122p3U was provided to site 1, WT as well as each of the RAVs were able to accumulate to some extent in miR-122 KO cells (Fig. 2*D* and *SI Appendix*, Fig. S1*C*). Interestingly, while C3U accumulated to a similar extent as WT, the G28A and C37U RAVs accumulated to a greater extent than WT, while the U4C and C2GC3U RAVs accumulated to a lesser extent. In contrast, complementation of site 2 allowed for the greatest accumulation of G28A followed by similar levels of accumulation of the WT, C2GC3U, and U4C RAVs, while the C3U and C37U RAVs were substantially impaired under these conditions (Fig. 2*E* and *SI Appendix*, Fig. S1*D*). Taken together, these results suggest that the G28A RAV is the most fit in the absence of miR-122, while the remaining RAVs can compensate for the loss of miR-122 at least at one of the two miR-122–binding sites. Moreover, the differential ability of the RAVs to accumulate either in the absence of miR-122 or when complemented specifically at site 1 or site 2, prompted us to further investigate the mechanism(s) of action of the RAVs.

### Several RAVs Alter the Structure of the 5′ UTR of the Positive-Strand Viral RNA.

**Fig. 3.**
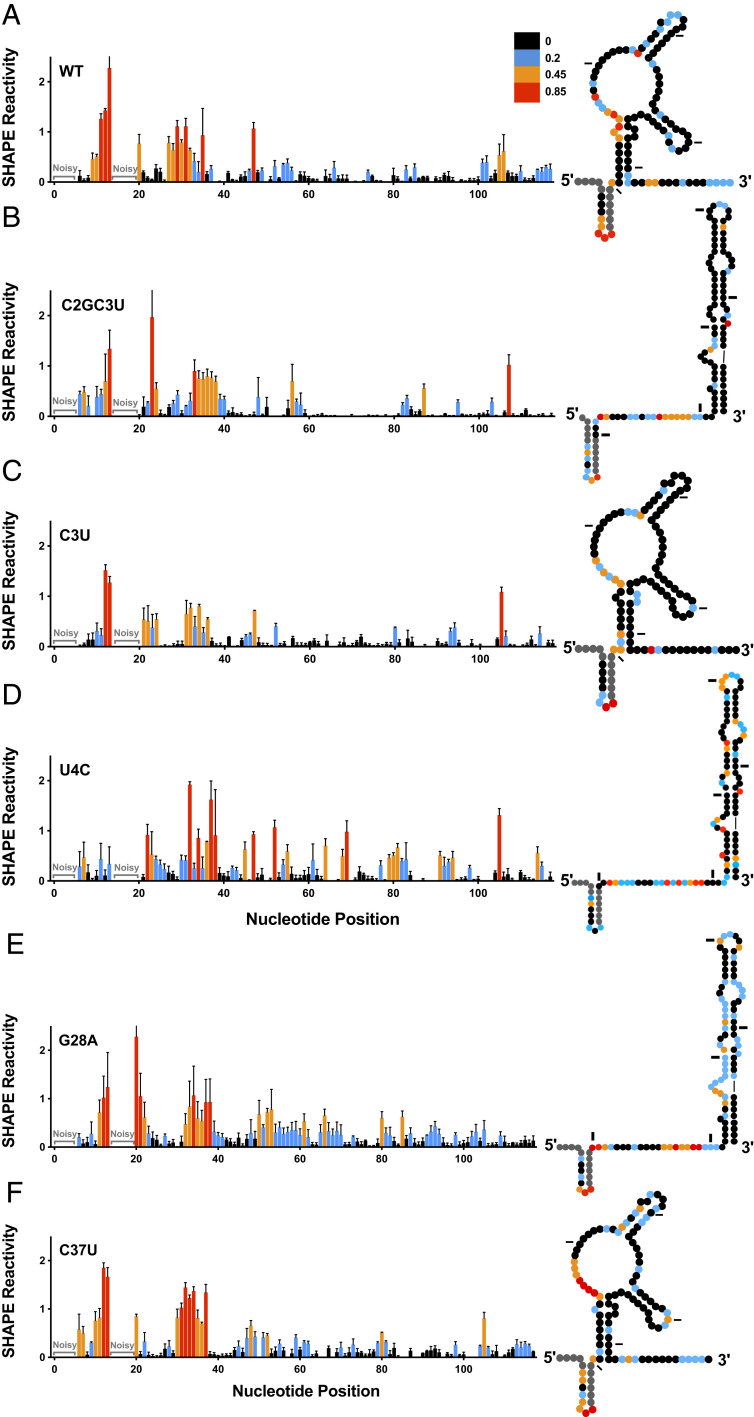
In vitro SHAPE analysis of viral RNAs suggests RAVs alter the structure of the viral RNA in the absence of miR-122. Normalized SHAPE reactivities of nucleotides 1 to 117 of (*A*) WT, (*B*) C2GC3U, (*C*) C3U, (*D*) U4C, (*E*) G28A, and (*F*) C37U HCV RNAs. Nucleotides 1 to 5 and 14 to 19 were omitted due to high background reactivity. Nucleotides with high (≥0.85, red), intermediate (between 0.4 and 0.85, orange), low (between 0.2 and 0.4, blue), and very low (≤0.2, black) SHAPE reactivity are indicated. The SHAPE reactivity data are superimposed on dot plots representative of the structural prediction that best fits the SHAPE reactivity of nucleotides 1 to 117 of the viral RNAs (*Right*). Tick marks represent 20 nucleotide intervals. The data are representative of three or more independent replicates, and error bars represent the SEM.

In contrast to WT, several recent studies have suggested that the G28A RAV is able to form the functional SLII structure, even in the absence of miR-122 (16, 17). Thus, we performed RNA structure prediction and in vitro selective 2′-hydroxyl acylation analyzed by primer extension (SHAPE) analysis to determine whether the RAVs alter the secondary structure of the viral 5′ UTR (*SI Appendix*, Fig. S2 and Fig. 3). We performed in vitro SHAPE analysis followed by capillary electrophoresis on the 5′ UTR (nucleotides 1 to 371) of the WT HCV RNA as well as each of the RAVs, and the results for nucleotides 1 to 117 (which includes SLI and SLII) are presented in Fig. 3. As demonstrated previously, in the absence of miR-122, the WT HCV RNA adopts an alternative, more energetically favorable structure, termed SLII^alt^ (Fig. 3*A* and *SI Appendix*, Fig. S2*A*) (15–17). Interestingly, in agreement with RNA structure predictions, several of the RAVs, including C2GC3U, U4C, and G28A, are essentially riboswitched and adopt the functional SLII structure, even in the absence of miR-122 (Fig. 3 *B*–*F* and *SI Appendix*, Fig. S2 *B*–*F*). Like WT, the C3U and C37U RAVs favored the alternative (SLII^alt^) structure (Fig. 3 *C* and *E*). However, although the most energetically favorable predicted structure of C3U and G28A is the alternative (SLII^alt^) structure, there is a very close equilibrium between the two structures (*SI Appendix*, Fig. S2 *C* and *E*), which is reflected in our in vitro SHAPE analysis, particularly at nucleotides 20 to 23 and 30 to 37 (Fig. 3 *C* and *E*). Since SHAPE reactivity reflects an average of the structures that form in solution, the difference in reactivity at these nucleotides when compared with WT suggests that the C3U and G28A RAVs are likely to exist in a close equilibrium between the alternative and functional structures. Finally, SHAPE analysis of C37U agreed with the RNA structure prediction, suggesting that C37U favors the alternative structure (SLII^alt^) similar to WT HCV RNA (Fig. 3*F* and *SI Appendix*, Fig. S2*F*).

In addition to the riboswitched conformation, we also noticed other subtle changes in RNA structure that might have functional consequences for the RAVs. Specifically, we noted that the C2GC3U and U4C RAVs had additional base-pairing interactions at the 5′ terminus that reduce the number of single-stranded nucleotides at the 5′ end of the viral RNA (Fig. 3 *A*–*D*). For C3U, although not represented in the SHAPE data, likely due to the close equilibrium between the SLII^alt^ and functional SLII conformations, structural prediction analysis suggests that this mutation also has the potential to increase base-pairing interactions at the 5′ terminus, reducing the single-stranded nucleotides at the 5′ end of the viral RNA (*SI Appendix*, Fig. S2*C*). Thus, our structure prediction and SHAPE analyses suggest that the RAVs can be generally categorized into three overlapping groups based on changes to the structure of the viral RNA. Specifically, the C2GC3U, U4C, and G28A RAVs are functionally “riboswitched,” even in the absence of miR-122. The C2GC3U, C3U, and U4C RAVs all have additional base-pairing interactions at the 5′ terminus of the HCV genome. And finally, the C37U RAV does not appear to alter the structure of the 5′ UTR of the positive-strand viral RNA.

### C2GC3U, U4C, and G28A Are Riboswitched, Even in the Absence of miR-122.

To provide further evidence that C2GC3U, U4C, and G28A are riboswitched in the absence of miR-122, we used electrophoretic mobility shift assay (EMSA) to explore the binding of hAgo2:miR-122 complexes to 1 to 117 nucleotide HCV RNAs (Table 1 and *SI Appendix*, Fig. S3). We predicted that due to their riboswitched conformation, the C2GC3U, C3U, and U4C RAVs may have a slightly higher affinity for hAgo2:miR-122 complexes because they exist in a more open conformation. For WT 1 to 117 nucleotide HCV RNA, we predict site 2 likely has a higher affinity and is bound first followed by a binding to site 1 based on the SLII^alt^ conformation. In agreement with this, we observed dissociation constants of 33 ± 10 pM (*K*_*d*_*1*) and 277 ± 130 pM (*K*_*d*_*2*) in line with previous measurements of hAgo2:miR-122 complex interactions with HCV RNAs (16). Importantly, the introduction of a mutation into site 2 (S2:p3) resulted in reduced binding (Table 1 and *SI Appendix*, Fig. S4). In line with our predictions, the C2GC3U and U4C RAVs had slightly higher affinities for site 2 binding (*K*_*d*_*1*) and reduced binding to site 1 (*K*_*d*_*2*) (Table 1). While for G28A, we observed similar binding affinities to WT for both sites (although we note that the SE was particularly large for G28A). Nonetheless, the results from EMSA analyses support our conclusion from SHAPE analysis that C2GC3U, U4C, and G28A are riboswitched, even in the absence of miR-122.

**Table 1.** Binding analysis of hAgo2:miR-122 to C2GC3U, C3U, and U4C RAVs

HCV RNA (nt)	WT ([nM] ± SE)*		S2:p3 ([nM] ± SE)*	
	*K*_*d*_ *1*	*K*_*d*_ *2*	*K*_*d*_ *1*	*K*_*d*_ *2*
WT (1-117)	0.033 ± 0.010	0.277 ± 0.130	0.017 ± 0.003	4.56 ± 6.57
C2GC3U (1-117)	0.019 ± 0.003	13.4 ± 56.4	2.34 ± 46.9	40.2 ± 812
U4C (1-117)	0.022 ± 0.016	0.413 ± 0.508	0.053 ± 0.011	2.91 ± 6.90
G28A (1-117)	0.036 ± 0.032	0.195 ± 0.261	0.022 ± 0.004	1.75 ± 2.05

* Values reported are an average of three independent measurements.

### Altered Base Pairing at the 5′ Terminus of C2GC3U and U4C Confers Viral RNA Stability.

**Fig. 4.**
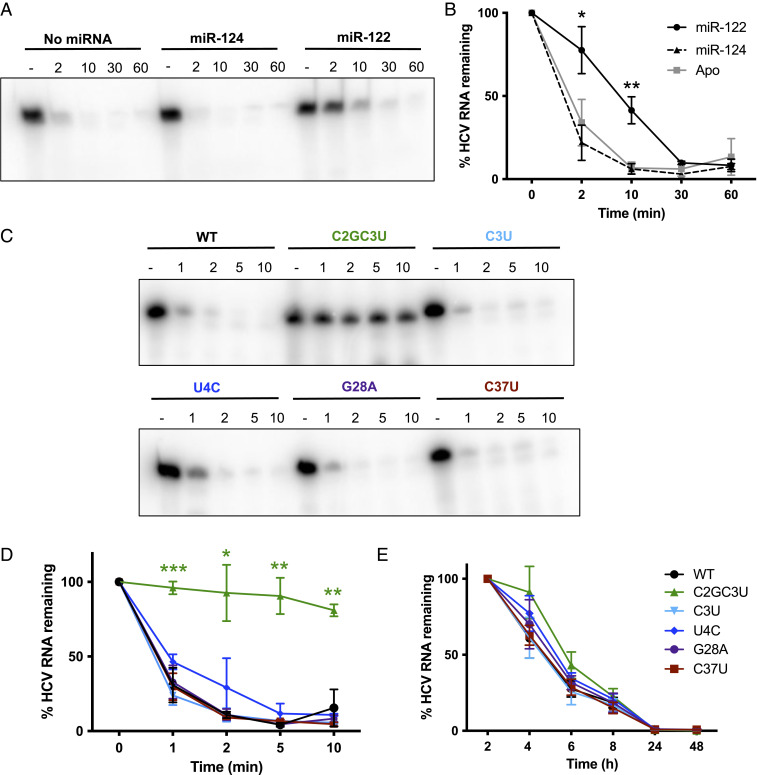
miR-122 and the C2GC3U and U4C RAVs provide stability to the HCV genome. (*A*) Xrn-1 assay with ^32^P-end–labeled monophosphorylated WT HCV RNA (nucleotides 1 to 42) incubated with no microRNA or the guide strand of miR-124 (negative control) or miR-122 incubated for 2 to 60 min (as indicated). (*B*) Quantification of the results in *A* graphed as percentage HCV RNA remaining over time. (*C*) Xrn-1 assay with ^32^P-end–labeled monophosphorylated WT and RAV HCV RNAs (nucleotides 1 to 42) incubated for 1 to 10 min (as indicated). (*D*) Quantification of the results in *C* graphed as percentage HCV RNA remaining over time. (*E*) miR-122 KO cells were coelectroporated with full-length RLuc S2:p3 GNN (replication-defective) HCV RNAs, a capped FLuc mRNA, and compensatory miR-122p3U. Luciferase activity was measured at the indicated time points post-electroporation. Viral RNA decay is represented as percentage HCV RNA remaining based on the luciferase activity at the 2-h time point. All data are representative of three independent replicates, and error bars represent the SD of the mean. Statistical significance was determined by multiple Student’s *t* test, ***P ≤ 0.001 and **P ≤ 0.01, *P ≤ 0.05.

Previous studies have suggested that miR-122 protects the HCV genome from two cellular pyrophosphatases, decapping exonuclease (DXO, also known as Dom3Z) and dual specificity phosphatase 11 (DUSP11) as well as subsequent exoribonuclease-mediated decay by Xrn-1 and/or 2 (14, 19–21, 27, 28). Thus, we hypothesized that base-pairing interactions between miR-122 and the 5′ terminus of the HCV genome could shield the 5′ terminus from pyrophosphatase and/or exoribonuclease activities. To explore this further, we performed in vitro exoribonuclease assays using Xrn-1 and nucleotides 1 to 42 of the HCV genomic RNA in the presence or absence of miR-122 or a negative control miRNA, miR-124 (Fig. 4 *A* and *B*). Our results suggest that in the absence of miR-122, the viral RNA is quickly degraded but that miR-122 base-paring interactions provide stability to the viral RNA from Xrn-1–mediated decay.

Based on our RNA structure predictions and in vitro SHAPE analysis, the C2GC3U, C3U, and U4C RAVs were shown or predicted to have additional base-pairing interactions at the 5′ terminus of the HCV genome (Fig. 3 *B*–*D* and *SI Appendix*, Fig. S2 *B*–*D*). Thus, we hypothesized that this additional base pairing could provide stability to these RAVs, even in the absence of miR-122. To investigate this, we performed in vitro exoribonuclease assays with each of the RAVs (Fig. 4 *C* and *D*). Interestingly, the C2GC3U RAV, which reduces the number of single-stranded nucleotides at the 5′ terminus of the HCV genome to 1 nt, substantially stabilizes the viral RNA (Fig. 4 *C* and *D*). While U4C, which converts a G-U wobble base pair to a C-G Watson–Crick pair at the base of SLI (Fig. 2*A*), also slightly stabilized the viral RNA (Fig. 4 *C* and *D*). The C3U mutation, which is predicted to provide only one additional base-pairing interaction (*SI Appendix*, Fig. S2), as well as the G28A and C37U RAVs, that do not alter base pairing at the 5′ terminus, had a similar stability to WT HCV RNA (Figs. 3 and 4 *C* and *D*). These results are not altogether surprising, as we observed that a two-nucleotide single-stranded 5′ overhang was required for Xrn-1 to initiate exoribonuclease activity and that Xrn-1 activity was more efficient as we increased the length of the single-stranded 5′ overhang (*SI Appendix*, Fig. S5). This is also in agreement with a previous study that suggests Xrn-1 requires a single-stranded 5′ overhang that is sufficiently long to reach the active site of the enzyme (29).

To further demonstrate whether this increase in base pairing at the 5′ terminus of the HCV genome results in increased stability of the viral RNA in cell culture, we performed viral RNA stability assays in miR-122 KO cells (Fig. 4*E* and Table 2). Specifically, we coelectroporated full-length Renilla luciferase (RLuc) S2:p3 GNN (replication-defective) reporter HCV RNAs and compensatory miR-122p3U molecules and followed viral RNA decay by luciferase assay. This experimental set up allows us to compensate for both the riboswitch and translational promotion activities provided by the site 2–bound miR-122 and thus directly assess viral RNA stability in the absence of site 1–bound miR-122. In agreement with our results in vitro, the C2GC3U and U4C RAVs had significantly increased half-lives (t_1/2_ = 3.5 and 2.9 h, respectively), when compared to WT HCV RNA (t_1/2_ = 2.4 h) in miR-122 KO cells (Fig. 4*E* and Table 2). The remaining RAVs, including C3U and C37U, had similar half-lives to WT; however, the G28A RAV demonstrated a slight increase in stability over WT HCV RNA (t_1/2_ = 2.7 versus 2.4 h, respectively). Taken together, our findings suggest that the increase in base pairing at the 5′ terminus of the HCV genome provided by the C2GC3U and U4C RAVs provides HCV genome stability, even in the absence of site 1–bound miR-122.

**Table 2.** Half-life of viral RNAs in cell culture

HCV	Half-life (h)*	95% CIs
WT	2.394	2.15 to 2.741
C2GC3U	3.519	2.828 to 4.658
C3U	2.308	2.016 to 2.701
U4C	2.946	2.541 to 3.503
G28A	2.679	2.296 to 3.215
C37U	2.383	2.173 to 2.637

* Values reported are an average of three independent measurements.

### C37U Alters the Structure of the 3′ End of the Negative-Strand Replicative Intermediate.

**Fig. 5.**
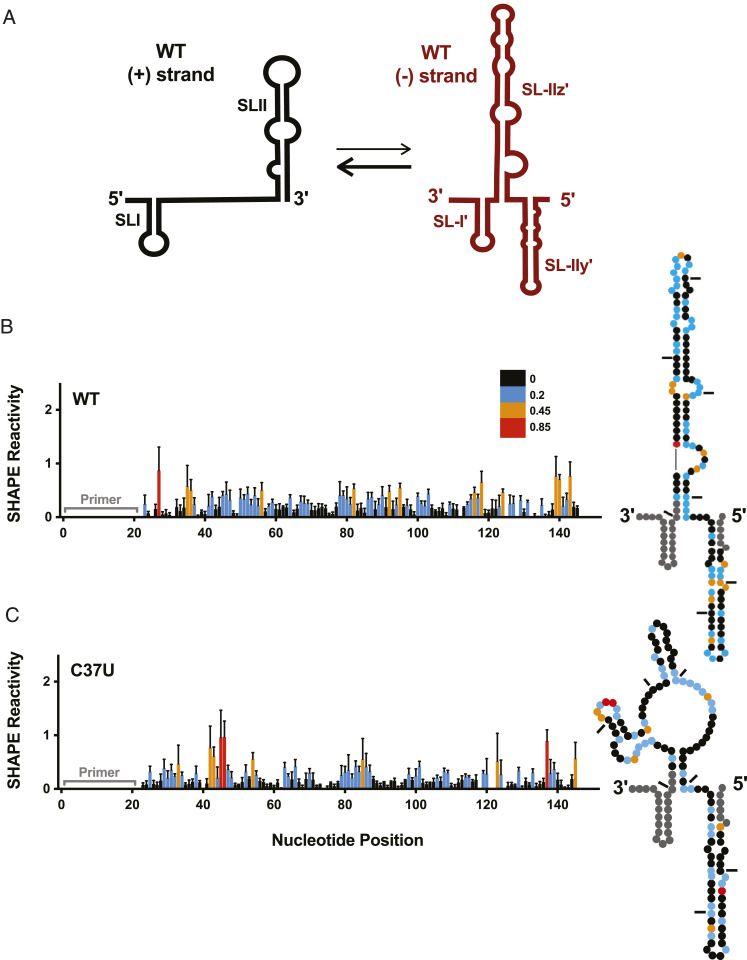
C37U alters the structure of the positive-strand promoter. (*A*) A model of the 5′ terminus of the positive-strand genomic RNA and the complementary 3′ terminus of the negative-strand RNA intermediate. Normalized SHAPE reactivities of nucleotides 1 to 151 of the 3′ terminus of the negative-strand RNA intermediate of (*B*) WT and (*C*) C37U numbered from the 3′ end. Nucleotides 1 to 19 and 149 to 151 were omitted, as the former corresponds to the primer binding site, and the latter demonstrated high SHAPE reactivity at the end of the transcript. Nucleotides with high (≥0.85, red), intermediate (between 0.4 and 0.85, orange), low (between 0.2 and 0.4, blue), and very low (≤0.2, black) SHAPE reactivity are indicated. The SHAPE reactivity data are superimposed on dot plots representative of the structural prediction that best fits the SHAPE reactivity of nucleotides 1 to 151 of the 3′ terminus of the negative-strand RNA intermediate (*Right*). Tick marks represent 20 nucleotide intervals. The data are representative of three or more independent replicates, and error bars represent the SEM.

Our RNA prediction and SHAPE analyses suggested that the C37U RAV did not alter the secondary structure of the viral 5′ UTR; thus, we hypothesized that this RAV might alter base pairing in the 3′ terminus of the negative-strand replicative intermediate, which adopts several stem–loop structures that are required for positive-strand RNA synthesis (5). Specifically, the 3′ terminus of the negative-strand replicative intermediate adopts three stem–loop structures, termed SL-I', SL-IIz', and SL-IIy' (Fig. 5*A*). SL-I' and SL-IIz' were previously demonstrated to be absolutely required for positive-strand RNA synthesis, while SL-IIy' was shown to improve the efficiency of positive-strand RNA synthesis (5). When we performed RNA structure prediction analyses, all of the other RAVs were predicted to adopt a WT-like conformation; however, the C37U RAV was predicted to adopt an alternative conformation (*SI Appendix*, Fig. S6), which we confirmed in solution using in vitro SHAPE analysis (Fig. 5 *B* and *C*). These results suggest that the C37U RAV alters the structure of the 3′ terminus of the negative-strand replicative intermediate, which represents the promoter for viral positive-strand (genomic) RNA synthesis.

### C37U Promotes Positive-Strand RNA Synthesis.

**Fig. 6.**
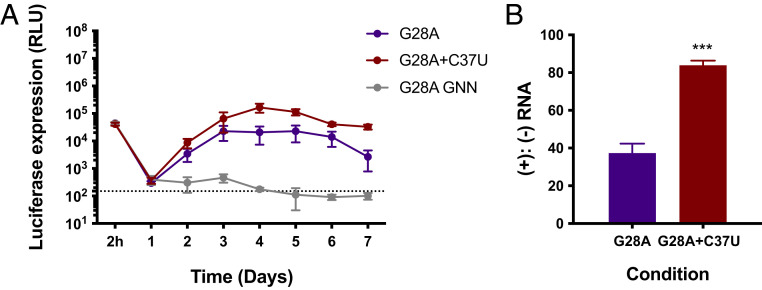
C37U promotes positive-strand RNA synthesis. (*A*) Full-length RLuc HCV genomic reporter RNAs (G28A, G28A+C37U, and G28A GNN) were coelectroporated with a capped Firefly luciferase mRNA into miR-122 KO cells. Luciferase activity was measured at the indicated time points post-electroporation. The limit of detection is indicated. (*B*) RT-qPCR analysis of G28A and G28A+C37U accumulation in miR-122 KO cells presented as the ratio of positive to negative (+):(−) strand RNA. Data are representative of three independent replicates, and error bars represent the SD of the mean. Statistical significance was determined by multiple Student’s *t* test, ***P ≤ 0.001.

To understand the impact of alteration of the structure of the 3′ terminus of the negative-strand RNA intermediate, we introduced the C37U mutation into the G28A RAV (which we previously demonstrated to be able to accumulate in the absence of miR-122, Fig. 2*C*) and we investigated viral RNA accumulation in miR-122 KO cells (Fig. 6). As we previously showed, the G28A RAV was able to accumulate in miR-122 KO cells, and the addition of the C37U mutation (G28A+C37U) resulted in even greater viral RNA accumulation (Fig. 6*A*). To investigate whether this was due to the alterations in the structure of the negative-strand intermediate resulting in the promotion of viral positive-strand synthesis, we performed strand-specific RT-qPCR analysis at the peak of replication (Fig. 6*B* and *SI Appendix*, Fig. S7). We observed that while G28A had a positive to negative strand ratio of 37.4, combining G28A+C37U resulted in a higher positive to negative strand ratio of 83.8 at day 4 post-electroporation. Notably, combining the U4C+C37U RAVs also rescued replication of U4C in miR-122 KO cells (*SI Appendix*, Fig. S8). Taken together, these results suggest that the C37U RAV alters the structure of the 3′ terminus of the negative-strand replicative intermediate in a manner that promotes positive-strand RNA synthesis.

## Discussion

**Fig. 7.**
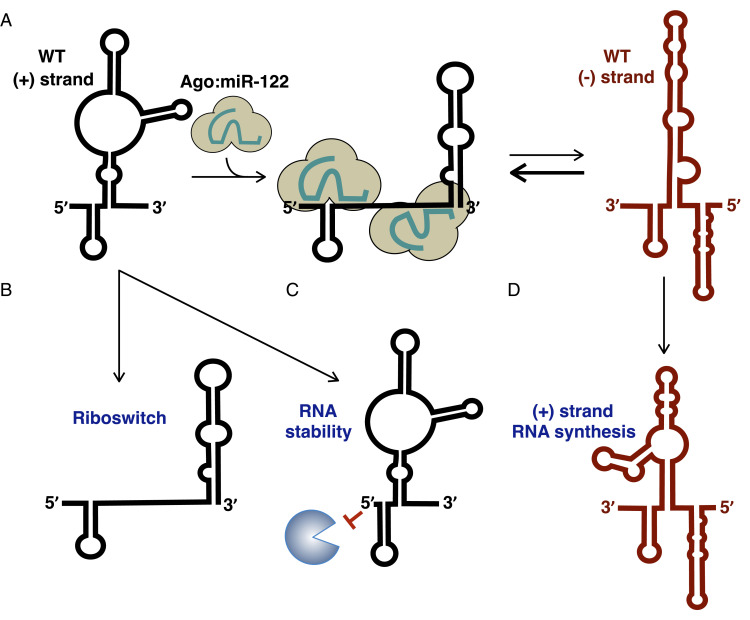
Mechanism of action of RAVs. (*A*) miR-122 promotes riboswitch, RNA stability, and translation of the WT positive-sense HCV RNA genome. (*B*) The C2GC3U, U4C, and G28A RAVs are riboswitched a priori, even in the absence of miR-122. (*C*) Alterations in base pairing at the 5′ terminus of the C2GC3U and U4C RAVs helps to stabilize the viral RNA, even in the absence of miR-122. (*D*) The C37U RAV alters the structure of the negative-strand RNA intermediate, which promotes positive-strand RNA synthesis.

Our analysis of miR-122 inhibitor RAVs has revealed three distinct mechanisms of action, all based on alterations to the structure of the viral RNA. More specifically, we found that 1) the C2GC3U, U4C, and G28A RAVs are riboswitched, even in the absence of miR-122; 2) the C2GC3U and U4C RAVs have increased base-pairing interactions at the 5′ terminus of the HCV genome that provide stability in the absence of miR-122; and 3) the C37U RAV alters the structure of the 3′ terminus of the negative-strand RNA intermediate, which promotes positive-strand RNA synthesis (Fig. 7).

Our findings suggest that, with the exception of U4C, the RAVs are collectively less fit than WT in the presence of miR-122, and only G28A is able to accumulate in the complete absence of miR-122. Interestingly, G28A was previously shown to be riboswitched (15–17) and to have an increased affinity for miR-122 (25); however, this RAV only had a fitness advantage when compared with WT when miR-122 was absent or limiting (i.e., complemented either at site 1 or site 2) (Fig. 2 and *SI Appendix*, Fig. S1). We suspect that this is likely explained by the close equilibrium that still occurs between the alternative (SLII^alt^, ∆G = −37.9) and functional (SLII, ∆G = −37.8) G28A structures when compared with the C2GC3U and U4C RAVs (*SI Appendix*, Fig. S2). Alternatively, this might also be due to a slight increase in affinity for Ago:miR-122 complexes at both sites, although our binding affinity measurements herein were not robust enough to reveal a significant difference in binding affinity when compared with WT HCV RNA. Specifically, since the G28A mutation converts a potential G-U wobble base pair to a A-U Watson–Crick base pair at this position, this likely provides an advantage in terms of recruiting miR-122 to site 2 but could result in a reduction in recruitment of a second Ago:miR-122 complex to site 1. In addition, human Ago2 possesses a solvated surface pocket that specifically binds adenine nucleobases in the 1 position (t1) of target RNAs that can help anchor Ago2 to t1A-containing targets (30–32). This finding suggests that, in addition to an increased affinity of miR-122 to site 2, the G28A mutation is also likely to increase the overall affinity of the Ago2:miR-122 complex to site 1 of the HCV genome. These increases in affinity at both site 1 and site 2 explain our findings under miR-122 replete conditions since the initial recruitment to either site 1 or site 2 (since G28A is riboswitched) would preclude interactions with the second Ago2:miR-122 complex. This also explains the results we obtained under miR-122 limiting conditions at both sites since G28A would be better able to recruit miR-122 to either site individually when compared with WT HCV RNA. Moreover, this might also explain the slight increase in half-life of the G28A RAV we observed when only site 2 was complemented (Table 2 and Fig. 4*E*), as more efficient recruitment of Ago:miR-122 to site 2 may enhance ribosome association, thereby stabilizing the viral RNA. Notably, the vast majority of HCV isolates (∼80%), with the exception of genotypes 1c, 2a-c/k, and 6a, already contain an A at position 28, and as such, the functional SLII conformation is predicted to be more energetically favorable, suggesting that the majority of HCV isolates are riboswitched a priori (*SI Appendix*, Figs. S9 and S10) (25).

As expected for the C2GC3U and U4C RAVs, which stabilize the viral RNA in the absence of miR-122, in the presence of miR-122, they accumulated to a lesser extent than WT, likely due to reduced auxiliary base-pairing interactions with site 1–bound Ago:miR-122 at the 5′ terminus of the HCV genome. However, the additional base pairing at the 5′ terminus did not afford them a significant viral RNA accumulation advantage over WT when either site was complemented. Nonetheless, in vitro stability assays as well as decay assays in cell culture suggest that they are indeed more stable than WT HCV RNA. This is likely explained by either a reduced ability to recruit Ago:miR-122 to site 1 or the increased stability of SLI of the HCV genome, which could interfere with the initiation of positive-strand RNA synthesis (i.e., by increasing the stability of SL-I' in the negative strand). Interestingly, the C3U RAV did not have a significant advantage, nor was it significantly more stable than WT, likely because it only creates one additional U-A Watson–Crick pair at the base of SLI.

Finally, the C37U RAV was particularly interesting because it converts a C-G Watson–Crick pair to a U-G wobble base pair in the seed sequence of site 2 (Fig. 2*A*), which is predicted to reduce its affinity for Ago:miR-122 complexes at this site (33). This explains its reduced accumulation in miR-122 replete conditions, while the improved efficiency of the positive-strand promoter explains its increased fitness over WT HCV RNA when site 1 is complemented. Interestingly, the Y-shaped structure that the C37U RAV creates in the negative-strand resembles the conformation of stem–loop A (SLA) of the related flaviviruses (e.g., Dengue and Zika viruses), a Y-shaped structure at the 5′ terminus of the viral genome known to be important for recruitment of the viral RNA-dependent RNA polymerase (34–37). Thus, it is possible that the Y-shaped structure created by the C37U RAV either has a higher affinity for the HCV RNA-dependent RNA polymerase or promotes the initiation step of viral RNA synthesis. This is supported by our finding that combining G28A (riboswitched) with C37U resulted in more positive-strand RNA synthesis than the G28A RAV alone in miR-122 KO cells. Notably, combining the U4C (stable) and C37U RAVs also conferred the ability to accumulate in miR-122 KO cells (*SI Appendix*, Fig. S8). These results are truly astounding because they suggest that the evolutionary pressure to maintain miR-122 interactions results in a fitness cost to the virus in terms of production of progeny genomic RNAs. Thus, while the C2GC3U, U4C, and G28A RAVs alter the structure of the positive-strand RNA in a manner that compensates for the loss of one or more of miR-122’s activities, the C37U RAV overcomes miR-122 inhibition by creating a stronger promoter for positive-strand RNA synthesis.

Importantly, our analysis herein was based on a genotype 2a (J6/JFH-1), and while the U4C, G28A, and C37U mutations were all identified in a genotype 2 background, C2GC3U was isolated from genotype 3 and 4 patients, and the C3U and A4C mutations were isolated from genotype 1 patients and replicons, respectively (23, 38). Interestingly, the C3U mutation has been observed in genotype 1 isolates and is predicted to adopt several additional alternative structures in this context, which may contribute to its resistance to miR-122 inhibitors (38). In agreement with the hypothesis posed herein, a previous study showed that in the context of genotype 1, the C3U mutation does provide a fitness advantage when compared with WT HCV RNA when miR-122 levels are limiting and that this is also related to increased stability in this context (39). In contrast, the C2GC3U RAV was isolated from genotype 3/4 patients and RNA structure predictions in this context suggest that it adopts a similar structure as the genotype 2a used herein (*SI Appendix*, Fig. S10). Thus, although both miR-122–binding sites are 100% conserved across HCV isolates, and the remainder of the 5′ terminus is very highly conserved (12), subtle nucleotide substitutions between genotypes may account for some of the nuance observed herein. Nonetheless, our findings provide an in-depth analysis of resistance to miR-122–based therapies and has revealed three unique mechanisms of resistance, all based on changes to the structure of the viral RNA.

Finally, what are the clinical implications for the use of miR-122 inhibitors in the future? Firstly, our results do not suggest that HCV can easily develop resistance because all of the RAVs were relatively unfit when compared with WT, and the RAVs were only detected in a few patients. Furthermore, RAVs were detected ≥8 wk after a single dose of the miR-122 inhibitor (RG-101), presumably when miR-122 levels were on the rise in chronic HCV-infected patients (23, 38). Moreover, even in the complete absence of miR-122, combining RAVs (G28A+C37U or U4C+C37U) still resulted in less accumulation in miR-122 KO cells than WT HCV RNA under miR-122 replete conditions. We suspect that this is likely because miR-122 has additional role(s) in the viral life cycle beyond its riboswitch, genome stability, and translational enhancement activities. Thus, miR-122 inhibitors may yet find a place in combination therapies, particularly for nonresponders or those with resistance to current antiviral regimens.

## Methods

### Cell Culture.

Huh-7.5 cells were kindly provided by C. M. Rice and miR-122 KO Huh-7.5 cells were kindly provided by M. Evans (24, 40). Both Huh-7.5 and miR-122 KO cells were maintained in Dulbecco’s minimal essential medium supplemented with 10% fetal bovine serum, 1% nonessential amino acids, and 200 µM L-glutamate (Wisent, Inc.).

### Plasmids, Viral RNAs, and Oligonucleotides.

The plasmid containing the full-length JFH-1_T_ isolate (Japanese Fulminant Hepatitis-1 isolate, genotype 2a), with three cell culture–adapted mutations, was provided by Rodney Russell, Memorial University, St. John’s, NL, Canada (41). Plasmids pJ6/JFH-1 FL RLuc WT and GNN bear full-length HCV sequences that consist of structural proteins from the J6 isolate and the nonstructural proteins from JFH-1 isolate and a RLuc reporter (42). The “GNN” mutant contains the indicated inactivating mutations in the viral polymerase GDD motif. The S1:p3 (C26A) and S2:p3 (C41A) as well as the RAVs (C2GC3U, C3U, U4C, G28A, C37U, and G28A+C37U) were generated by overlapping PCR and subcloned using the *EcoR1* and *Kpn1* restriction sites on the pJ6/JFH-1 WT and GNN plasmids.

To make full-length viral RNAs, all templates were linearized and in vitro transcribed as previously described (14). To generate capped Firefly luciferase (43) mRNA, the Luciferase T7 Control DNA plasmid (Promega) was linearized using *XmnI* and in vitro transcribed using the mMessage mMachine T7 Kit (Life Technologies) according to the manufacturer’s instructions.

For in vitro SHAPE analyses, the 5′ UTR (nucleotides 1 to 371) of the HCV genome was in vitro transcribed using the T7 RiboMAX Express Kit (Promega) followed by gel purification as previously described (16). For negative-strand RNA analysis, we synthesized a geneblock (Integrated DNA Technologies [IDT]) containing a T7 promoter, nucleotides 1 to 151 of the 3′ terminus of the WT JFH-1 negative-strand HCV RNA (5′ to 3′), immediately followed by a 67-nucleotide deletion variant of the hepatitis delta virus ribozyme (HDVr) (44), flanked by *EcoRI* and *BamHI* restriction sites. The geneblock was subcloned into pUC18 using *EcoRI/BamHI*, and the RAVs were generated using the QuikChange II XL Site-Directed Mutagenesis Kit (Agilent Technologies) according to the manufacturer’s instructions. After linearization with *BamHI*, in vitro transcription reactions were carried out as described above. Negative-strand transcripts were generated after self-cleavage by the HDVr concurrently with the transcription reaction (44), and subsequently, negative-strand RNAs with precise 3′ termini (151 nucleotides in length) were gel purified.

For Xrn-1 exoribonuclease activity assays, nucleotides 1 to 42 of the WT and each of the RAV HCV RNAs were synthesized by IDT (*SI Appendix*, Table S1). All microRNAs used in this study were synthesized by IDT (*SI Appendix*, Table S2).

### Electroporations.

Electroporations were carried out as previously described (14, 45). Briefly, 1.5 × 10^7^ cells in 400 µL cold sterile phosphate-buffered saline (PBS; Wisent) were coelectroporated with 10 µg WT, GNN, and RAV full-length HCV RLuc RNAs, 2 µg capped Firefly luciferase mRNA, and in some cases, 60 pmol of duplexed miR-122p3U. Cells were electroporated using 4 mm cuvettes at 270 V, 950 µF, and infinite resistance, optimized for the BioRad GenePulser XCell (BioRad). Cells were resuspended in 4.5 mL media and 500 µL per timepoint were plated in 12-well or 6-well plates for luciferase assays at 2 h to 7 d post-electroporation.

### Luciferase Assays.

Cells were washed with cold sterile PBS (Wisent) and harvested in 100 µL 1× Passive Lysis buffer (Promega). The Dual Luciferase Assay Reporter Kit (Promega) was used for all samples analyzed for both *Renilla* and Firefly luciferase activity according to the manufacturer’s protocol. Firefly luciferase was used to ensure similar electroporation efficiency across samples.

### In Vitro SHAPE Analysis and Capillary Electrophoresis.

In vitro SHAPE analysis was performed as previously described (16). For positive-strand RNA analysis, 1 pmol 6-FAM– or NED-labeled oligonucleotides (5′-6-FAM/NED-CGC CCG GGA ACT TAA CGT CTT-3′) were used for the SHAPE samples and the sequencing ladders, respectively. For negative-strand RNA intermediate analysis, 1 pmol 6-FAM– or NED-labeled oligonucleotides (5′-6-FAM/NED-ACC TGC CCC TAA TAG GGG CGA C-3′) were used for the SHAPE samples and the sequencing ladders, respectively. Capillary electrophoresis was performed at Plateforme d’Analyses Génomiques de l’Univeristé Laval on an ABI 3100 Genetic Analyzer. Raw fluorescence data were analyzed using the QuSHAPE software as previously described (16, 46).

### RNA Structure Prediction.

RNA structure predictions were carried out using the RNAstructure software, available from the Matthews Lab at https://rna.urmc.rochester.edu/index.html (48). Briefly, the RNA sequence was loaded into the RNAstructure software using the “Fold RNA Single Strand” command as previously described (16, 47).

### RNA Labeling.

In vitro transcribed HCV (1-117) RNAs were ethanol precipitated, resuspended in RNase-free H_2_O and treated with DNase I (New England Biolabs) to remove the DNA template, followed by purification with Sephadex G-25 spin columns (Cytiva) to remove unincorporated NTPs and the degraded template. The flowthrough was treated with quickCIP (New England Biolabs) to remove the 5′ triphosphate followed by heat inactivation of the phosphatase (2 min at 80 °C). Viral RNAs were labeled with T4 PNK (New England Biolabs) and γ[^32^P]ATP followed by gel purification on a denaturing (8 M urea) 15% acrylamide gel run in 0.5× Tris-borate-ethylenediaminetetraacetic acid (TBE) buffer. Bands were imaged, excised, and crushed in 300 µL crush and soak buffer (0.5 M NH_4_OAc, 0.2% sodium dodecyl sulfate, and 1 mM ethylenediaminetetraacetic acid [EDTA]) followed by elution at 4 °C overnight. The gel was removed with a spin-X column (Sigma Aldrich), and the RNA was precipitated using 3M NaOAc, pH 5.2 and ice-cold 100% ethanol at −20 °C for >20 min. RNAs were pelleted by centrifugation, washed with 150 µL ice-cold 75% ethanol, dried, resuspended in RNase-free H_2_O, and quantified by nanodrop spectrophotometer. Extinction coefficients were calculated with the IDT online tool available at https://www.idtdna.com.

### Stability Assays.

A total of 100 pmol monophosphorylated 1 to 42 nucleotide WT and RAV HCV RNAs (*SI Appendix*, Table S1) were end labeled using T4 RNA ligase and [γ-^32^P] ATP and purified using an RNA Clean & Concentrator-5 Spin Column (Zymo Research). A total of 2 pmol end-labeled RNA was refolded by denaturing at 95 °C for 3 min followed by incubation at 37 °C for 10 min. For experiments with miRNAs, 4 pmol of miR-122 or miR-124 (control) guide RNAs were added, and reactions were incubated for 20 min at 37 °C following the refolding step. Xrn-1 assays were carried out using 1 unit Terminator 5′-Phosphate-Dependent Exonuclease in 1× Terminator Reaction Buffer A (Lucigen) and 1 unit Ribolock (Life Technologies). The reactions were incubated at 30 °C and were quenched with 1 µL 100 mM EDTA after incubation for 1 to 60 min. At the indicated time points, 10 µL Gel Loading dye (Life Technologies) was added to each sample, and 5 µL each sample was resolved on a 15% denaturing polyacrylamide gel (29:1 acrylamide:bis-acrylamide, 1× TBE), dried at 80 °C for 1.5 h (BioRad Gel dryer, Model 583), and visualized by a phosphoimager (Storm, GE Life Sciences). Image analysis was performed using the ImageJ software. For live cell stability assays, viral RNA half-lives were calculated using GraphPad Prism by fitting the data to a one-phase exponential decay model.

### RT-qPCR Analysis.

Total RNA was harvested and extracted from cells using the TriZol reagent (Thermo Fisher Scientific) according to the manufacturer’s protocol. Strand-specific RT-qPCR was performed as previously described (48) with a few modifications (*SI Appendix*, *Supplemental Methods*).

## Supplementary Material

Supplementary File

## Data Availability

All study data are included in the article and/or *SI Appendix*.
