# Supplementary material for: miR-122–based therapies select for three distinct resistance mechanisms based on alterations in RNA structure
Source: Proc Natl Acad Sci U S A. 2021 Aug 12;118(33):e2103671118. doi: 10.1073/pnas.2103671118 (PMC8379925; doi:10.1073/pnas.2103671118)
Supplement: Supplementary File [file pnas.2103671118.sapp.pdf]

Supplementary Information

Supplementary Methods

In vitro Xrn-1 assay using duplex substrates

For Xrn-1 assays using duplex substrates, oligonucleotides were synthesized by IDT (Table S1). One hundred picomoles of the monophosphorylated single-stranded 25-nt RNA oligonucleotide was end-labeled using T4 RNA ligase and [γ-^{32}P] ATP and purified using an RNA Clean & Concentrator-5 spin column (Zymo Research). The 20-24-nt complementary DNA oligonucleotides were added to the end-labeled RNA (in a 2.5-fold molar excess of DNA to RNA), followed by denaturation at 65°C for 3 min and cooled to room temperature. Xrn-1 assays were carried out as described using one unit of Terminator 5'-Phosphate-Dependent Exonuclease in 1X Terminator Reaction Buffer A (Lucigen), and 1 unit of Ribolock (Life Technologies). The reactions were incubated at 30°C and quenched with 1 µL of 100 mM EDTA after incubation for 5 min. Ten microliters of Gel Loading dye (Life Technologies) was added to each sample and 5 µL of each sample was resolved on a 15% denaturing polyacrylamide gel (29:1 acrylamide:bis-acrylamide, 1X TBE), dried at 80°C for 1.5 h (BioRad Gel dryer, Model 583), and visualized by phosphoimager (Storm, GE Life Sciences). Image analysis was performed using the ImageJ software.

Electromobility shift assays

hAgo2:miR-122 complexes were prepared as described previously (1). Binding reactions were prepared in a final volume of 20 µl, with a final concentration of 0.01 nM 5'-radiolabeled RNA, and a final concentration of hAgo2:miR-122 ranging from 0-2 nM were prepared in reaction buffer (30 mM Tris pH 8.0, 100 mM KOAc, 2 mM $Mg(OAc)_2$, 0.5 mM TCEP, 0.005% NP-40) and

incubated for 45 min at room temperature. The reactions were supplemented with 4 μl native loading buffer (30% glycerol, 0.25% xylene cyanol, 0.25% bromophenol blue) and analyzed on a 6% acrylamide native gel in 0.5× TBE (45 mM Tris-borate, 1 mM EDTA, pH 8.3). The gel was dried for 1.5 h at 80 °C and imaged using a Typhoon phosphoimager (GE Healthcare Life Sciences).

To calculate the dissociation constant (K_d), equilibrium binding data of triplicate electrophoretic mobility shift assays were fit to following equations:

$$\text{Site 1} = \text{Bmax1}*[\text{hAgo2}]/(K_d1+[\text{hAgo2}])$$

$$\text{Site 2} = \text{Bmax2}*[\text{hAgo2}]/(K_d2+[\text{hAgo2}])$$

$$Y = \text{Site 1} + \text{Site 2}$$

where Y is the fraction of target RNA bound, Bmax1 (constrained to a maximum value of 1) is the calculated value of maximum binding for the high affinity K_d1, Bmax2 is the calculated value for the maximum binding for the low affinity K_d2 (constrained to a maximum value of 1), [hAgo2] is the total concentration of the hAgo2:miR-122 complex, and K_d1 and K_d2 are the calculated dissociation constants, obtained using Graphpad Prism. To quantify the data from the phosphorscreen, Bmax was normalized to 2: the total bound sites at each concentration were calculated as (2*[double shift intensity] + [single shift intensity]), and the free sites were calculated as ([single shift intensity] + 2*[unbound RNA intensity]).

Strand-specific reverse transcription quantitative polymerase chain reaction (RT-qPCR)

Total RNA was extracted from cells using the TriZol reagent (Thermo Fisher Scientific) according to the manufacturer's protocol. Strand-specific RT-PCR was performed using a thermostable polymerase and tagged strand-specific primers in the RT step, followed by

qPCR using a tag- and HCV-specific primer on the Bio-Rad CFX96 Touch Real-Time System, as previously described (2), with a few modifications. Briefly, 1 μg of total RNA was mixed with 5 μM deoxynucleoside triphosphate (dNTP) and 10 nM of a tagged strand-specific primer and incubated at 95°C for 5 minutes to destroy RNA secondary structures and to denature dsRNA. Then Maxima H Minus Reverse Transcriptase (ThermoFisher Scientific), RNAsin (Promega, Mannheim, Germany), and the standard buffer provided with the RT enzyme were added. The RT reaction was performed for 30 minutes at 55°C, with subsequent heat inactivation at 85°C for 15 minutes. The strand-specific primers used were **Tag5**_S146_JFH (**CTG GAG TCG TAG ATC CTA CCG C**TC TGC GGA ACC GGT GAG TA) or **Tag4**_A219_JFH (**GAA GCT GAC TTG ACA TGT TGC C**GG GCA TAG AGT GGG TTT ATC CA) for positive- and negative-strand detection of HCV isolate JFH-1 (2). The cDNA was then purified to remove excess RT primers using the Zymo DNA clean and concentrator (Zymo Research) according to manufacturer's instructions. The concentrated product was diluted 1:2 with RNase free water and used for qPCR with QuantiNova Probe PCR Kit (Qiagen, Hilden, Germany). The PCR primers used were Tag4 (GAA GCT GAC TTG ACA TGT TGC C), Tag5 (CTG GAG TCG TAG ATC CTA CCG C); S_146_JFH (TCT GCG GAA CCG GTG AGT A), A_219_JFH (GGG CAT AGA GTG GGT TTA TCC A). The TaqMan probes used were HCV-JFH1-Probe (6-FAM-AAA GGA CCC AGT CTT CCC GGC AAT T- TAMRA) and PrimePCR GAPDH primers and HEX probe (Bio-Rad). Viral RNAs were normalized to GAPDH using the ΔCt method and compared to a standard curve generated using either the positive or negative-strand *in vitro* transcribed JFH-1/J6 RNA. The relative amount of positive- and negative-strand viral RNAs were calculated using the $2^{(-\Delta\Delta Ct)}$ method as previously described (3). Viral RNA copy number was then used to calculate the ratio of positive- to negative-strand viral RNAs.

Supplementary References

1. J. Sheu-Gruttadauria, I. J. MacRae, Phase Transitions in the Assembly and Function of Human miRISC. *Cell* **173**, 946-957 e916 (2018).
2. O. Grunvogel *et al.*, Secretion of Hepatitis C Virus Replication Intermediates Reduces Activation of Toll-Like Receptor 3 in Hepatocytes. *Gastroenterology* **154**, 2237-2251 e2216 (2018).
3. K. J. Livak, T. D. Schmittgen, Analysis of relative gene expression data using real-time quantitative PCR and the 2(-Delta Delta C(T)) Method. *Methods* **25**, 402-408 (2001).
4. C. Combet *et al.*, euHCVdb: The European hepatitis C virus database. *Nucleic Acids Res.* **35** (2007).

Supplementary Tables

Table S1. Oligonucleotides used in Xrn1 assays.

Oligonucleotides	Sequence (5' to 3')
**WT 1-42**	ACC UGC CCC UAA UAG GGG CGA CAC UCC GCC AUG AAU CAC UCC
**C2GC3U 1-42**	AGU UGC CCC UAA UAG GGG CGA CAC UCC GCC AUG AAU CAC UCC
**C3U 1-42**	ACU UGC CCC UAA UAG GGG CGA CAC UCC GCC AUG AAU CAC UCC
**U4C 1-42**	ACC CGC CCC UAA UAG GGG CGA CAC UCC GCC AUG AAU CAC UCC
**G28A 1-42**	ACC UGC CCC UAA UAG GGG CGA CAC UCC ACC AUG AAU CAC UCC
**C37U 1-42**	ACC UGC CCC UAA UAG GGG CGA CAC UCC GCC AUG AAU UAC UCC
**25-nt ssRNA**	AAA AAA AAC CCC ACC ACC AUC ACU U
**25-nt DNA**	dAdAdG dTdGdA dTdGdG dTdGdG dTdGdG dGdGdT dTdTdT dTdTdT
**24-nt DNA**	dAdAdG dTdGdA dTdGdG dTdGdG dTdGdG dGdGdT dTdTdT dTdT
**23-nt DNA**	dAdAdG dTdGdA dTdGdG dTdGdG dTdGdG dGdGdT dTdTdT dT
**22-nt DNA**	dAdAdG dTdGdA dTdGdG dTdGdG dTdGdG dGdGdT dTdTdT
**21-nt DNA**	dAdAdG dTdGdA dTdGdG dTdGdG dTdGdG dGdGdT dTdT

Table S2. MicroRNAs.

**Guide Strand**	**Sequence (5' to 3')**
**miR-122 (WT)**	UGG AGU GUG ACA AUG GUG UUU GU
**miR-122p3U**	UGU AGU GUG ACA AUG GUG UUU GU
**miR-124**	UAA GGC ACG CGG UGA AUG CC
**Passenger Strand**	**Sequence (5' to 3')**
**miR-122***	AAA CGC CAU UAU CAC ACU AAA UA
**miR-122p3U***	AAA CGC CAU UAU CAC ACU CAA UA

Supplementary Figures

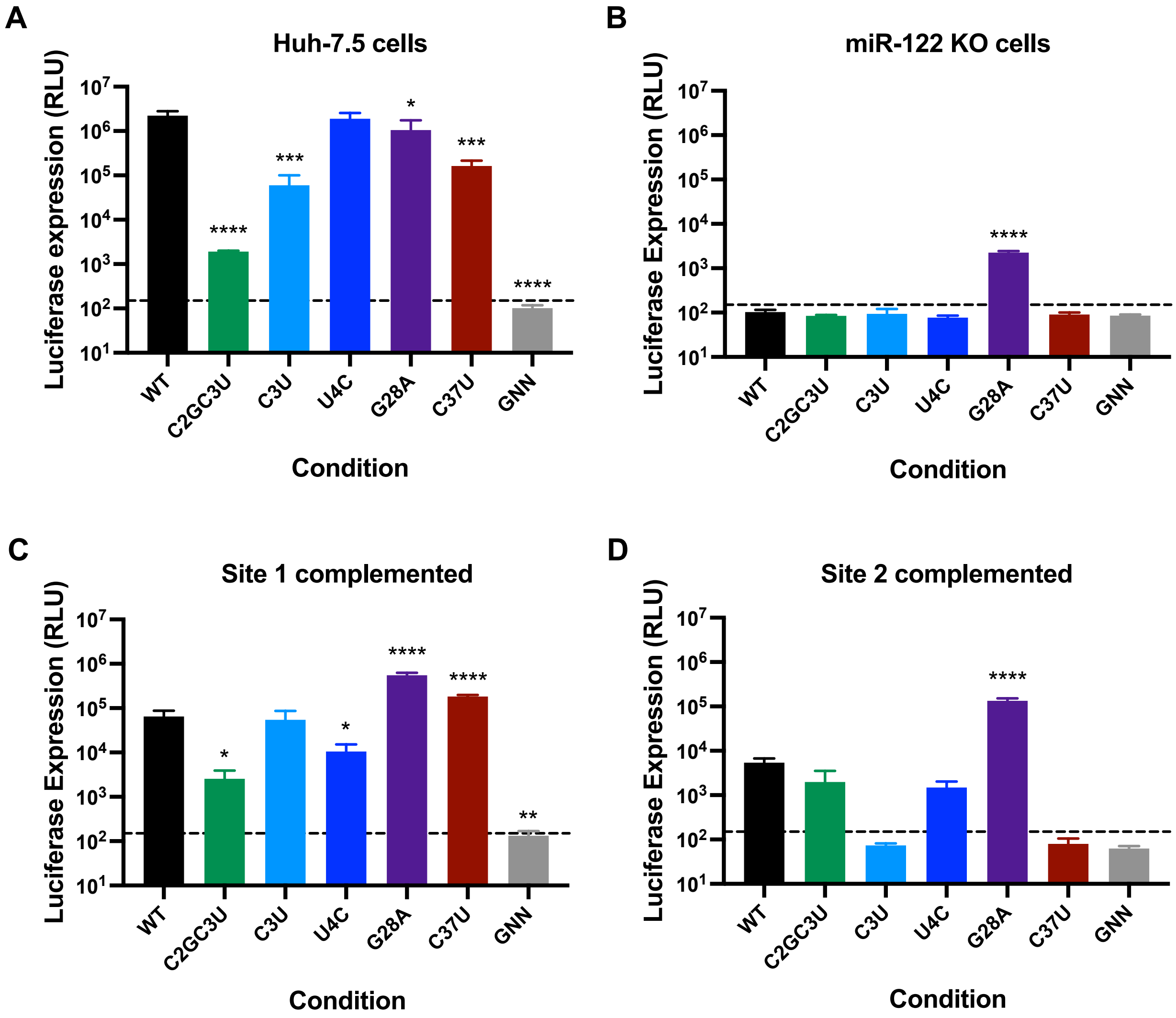

Figure S1. Statistical analysis of RAV accumulation in cell culture. Statistical analysis of RAV accumulation from Figure 2 at Day 2 post-infection in (A) Huh-7.5 cells and (B) miR-122 KO cells. Statistical analysis of RAV accumulation from Figure 2 at Day 2 post-infection when (C) Site 1 (S1:p3A) or (*D*) Site 2 (S2:p3A) were co-electroporated *with* compensatory miR-122p3U molecules into miR-122 KO cells. The limit of detection is indicated and all data are representative of three independent replicates. Error bars represent the standard deviation of the mean. Statistical significance compared with WT was determined by multiple t-test, ****$p \leq 0.0001$, ***$p \leq 0.001$, **$p \leq 0.01$, *$p \leq 0.05$. No labeling indicates not significant with respect to WT accumulation.

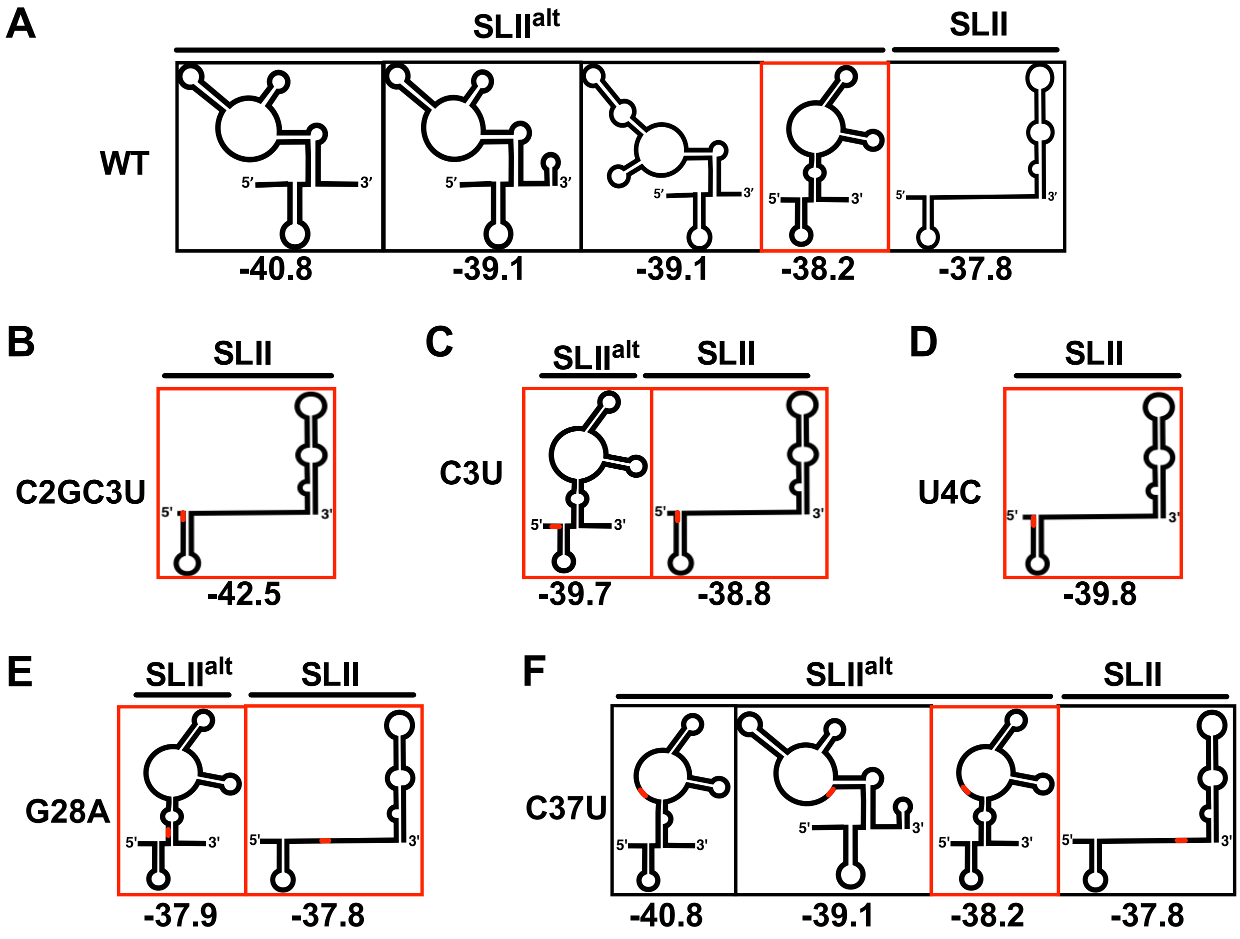

Figure S2. RNA structure predictions of the 5' UTR (nucleotides 1-117) of the positive-strand HCV genomic RNA. Alternative structural predictions and the predicted free energies (ΔG, kcal/mol) are indicated. **(A)** WT, **(B)** C2GC3U, **(C)** C3U, **(D)** U4C, **(E)** G28A and **(F)** C37U. The mutations in the HCV RNAs are shown in red. The structure best aligned with SHAPE analysis is highlighted in red.

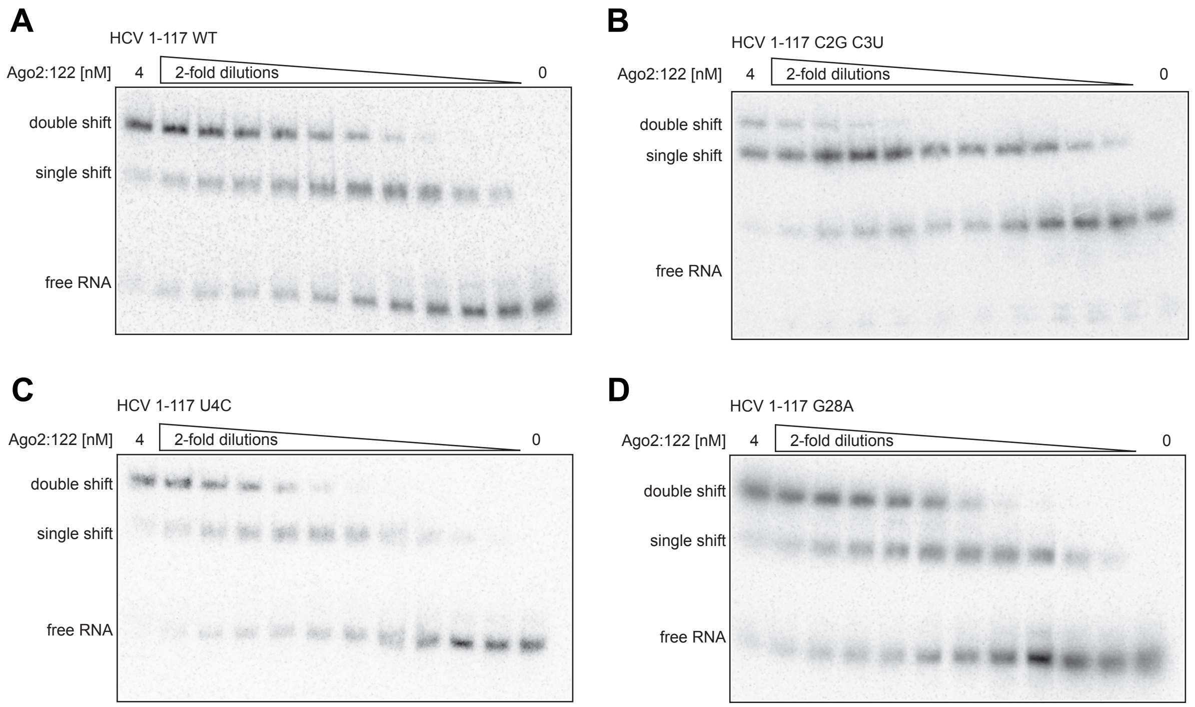

Figure S3. Electrophoretic mobility shift assays of WT and C2GC3U, C3U and U4C RAVs. Representative gels of the electrophoretic mobility shift assays with hAgo2:miR-122 and the 5' radiolabeled 1-117 nt RNAs from the 5' UTR of the (A) WT, (B) C2GC3U, (C) U4C, and (D) G28A HCV RNAs.

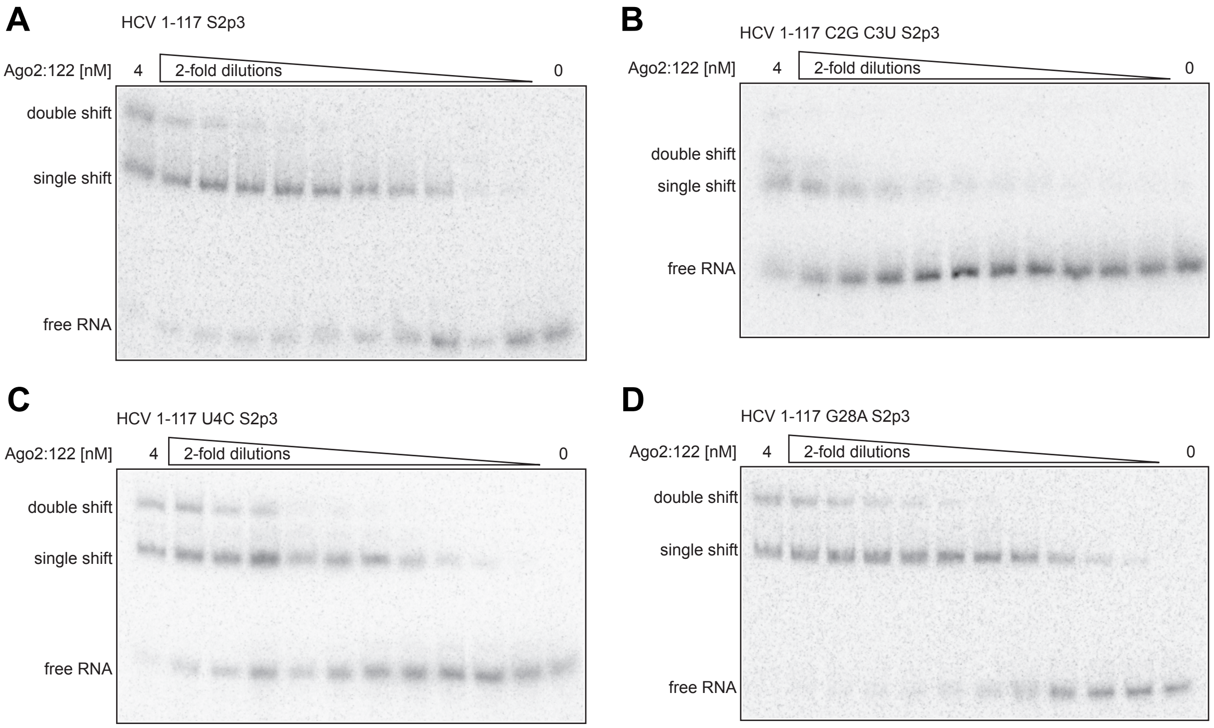

Figure S4. Electrophoretic mobility shift assays of WT and C2GC3U, C3U and U4C RAVs using S2:p3 mutant viral RNAs. Representative gels of the electrophoretic mobility shift assays with hAgo2:miR-122 and the 5' radiolabeled 1-117 nt S2:p3 RNAs from the 5' UTR of (A) WT, (B) C2GC3U, (C) U4C, and (D) G28A mutants of the HCV genome.

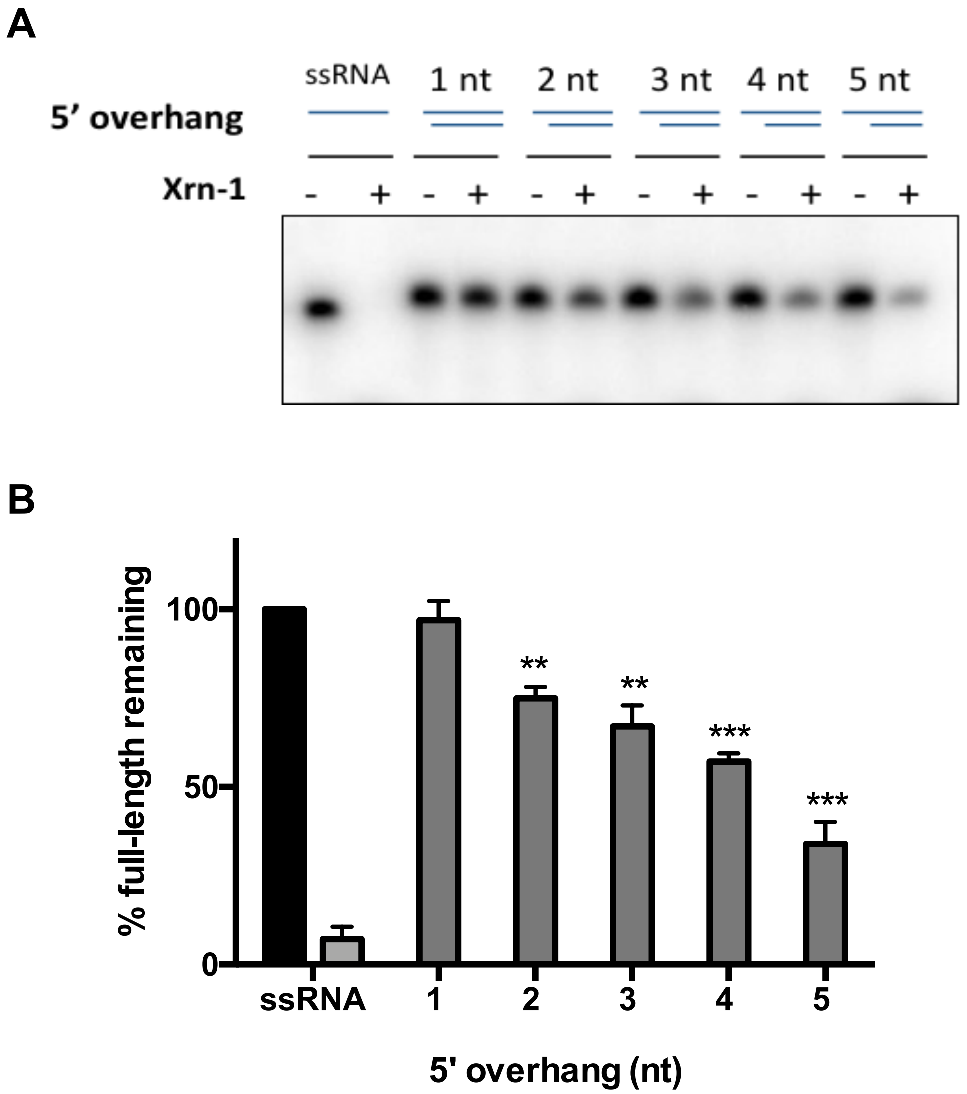

Figure S5. Xrn-1 requires several 5' single-stranded nucleotides to initiate exoribonuclease activity. (**A)** Xrn-1 assay with ^{32}P-end-labeled monophosphorylated single-stranded RNA oligos were hybridized to various length complementary DNA oligos, resulting in 5′ overhangs of 1-5 nucleotides. **(B)** Quantification of the results in **(A)** graphed as % full-length RNA remaining. All data are representative of three independent replicates and error bars represent the standard deviation of the mean. Statistical significance was determined by unpaired t-test, ***$p \leq 0.005$*,*** *$p \leq 0.0005$*.

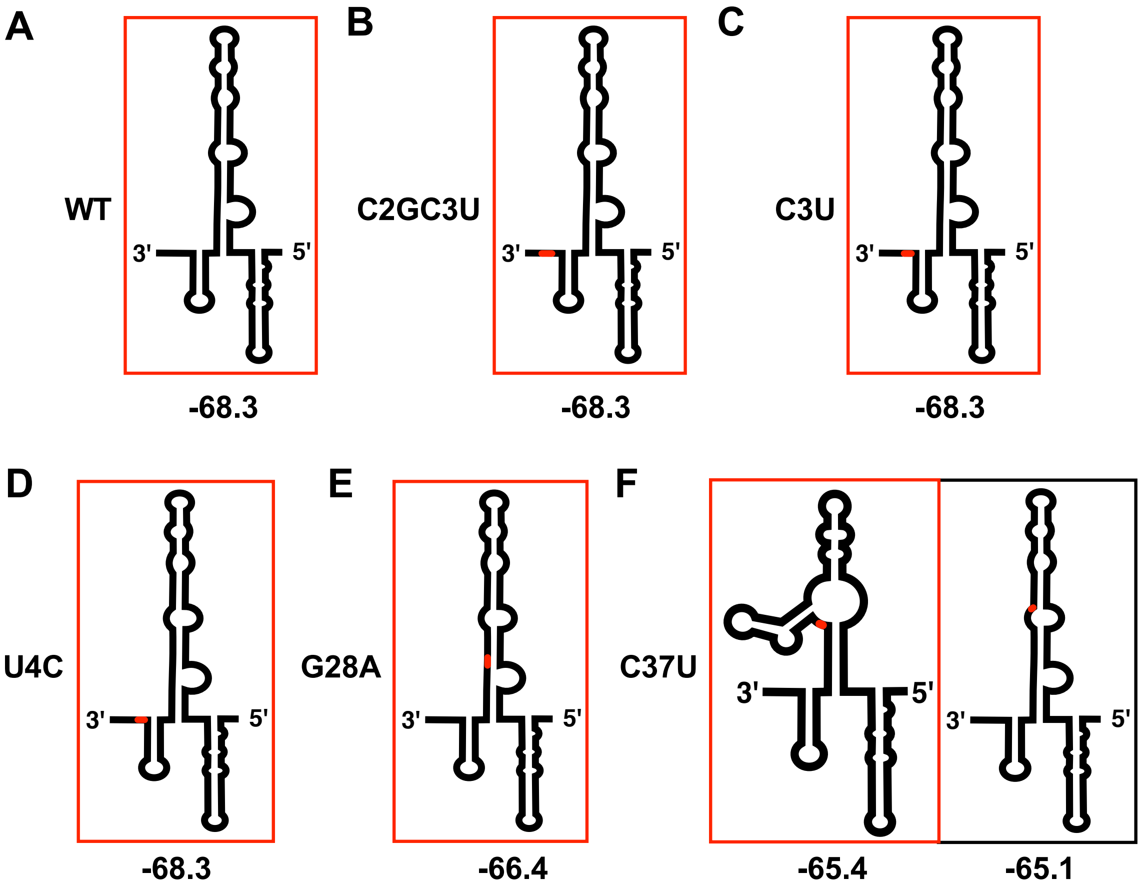

Figure S6. RNA structure predictions of the 3' end (nucleotides 1-151) of the negative-strand HCV genomic RNA. Alternative structural predictions and the predicted free energies (ΔG, kcal/mol) are indicated. **(A)** WT, **(B)** C2GC3U, **(C)** C3U, **(D)** U4C, **(E)** G28A and **(F)** C37U. The mutations in the HCV RNAs are shown in red. The most favourable structure based on RNA structure analysis (**B** through **E**) or with *in vitro* SHAPE analysis (**A** and **F**) is highlighted in red.

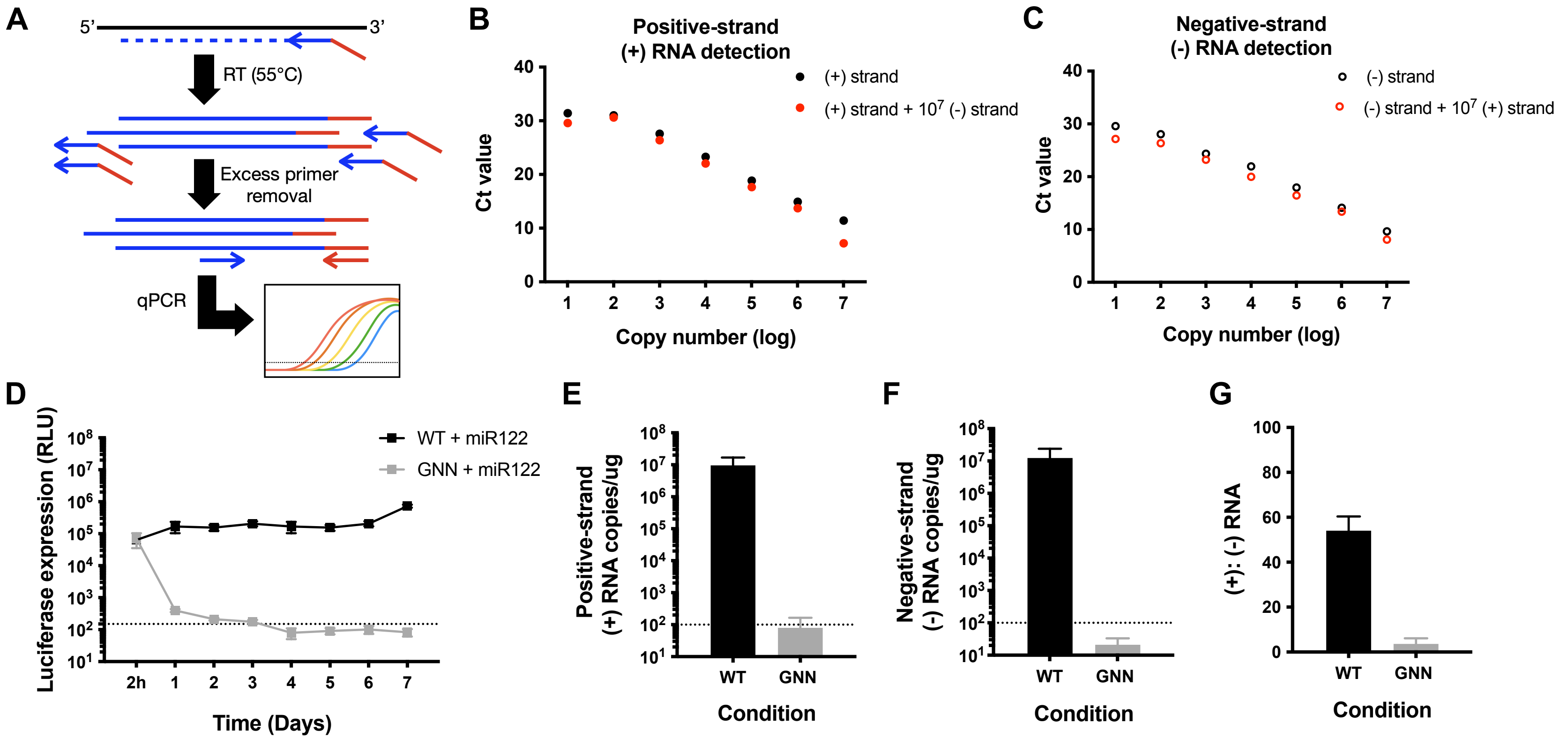

Figure S7. Strategy of strand-specific RT-qPCR assay. (A) Schematic representation of general strategy for strand-specific RT-qPCR. Briefly, RNA samples were heated to 95ºC for denaturation of secondary structures. RT was performed with a hot start at 55ºC to avoid non-specific priming of cDNA synthesis, using a tagged RT primer specific to the respective HCV strand. The tag is an arbitrary DNA sequence unrelated to the template shown in red, while the HCV-specific sequence is indicated in blue. After RT, the cDNA sample was cleaned up to eliminate excess RT primer. Specificity and sensitivity of strand-specific RT-qPCR initiated with (B) 10^7 of the positive-strand serially diluted ten-fold with or without 10^7 of the negative-strand spiked in or (C) 10^7 of the negative-strand serially diluted ten-fold with or without 10^7 of the positive-strand spiked in. The log of the RNA copies is plotted against the cycle threshold (Ct). (D) Full-length Renilla Luciferase (RLuc) HCV genomic reporter RNAs (WT and GNN) were co-electroporated with WT miR-122 and capped Firefly Luciferase (FLuc) mRNA into miR-122 KO cells. Luciferase activity was measured at the indicated time points post-electroporation. RT-qPCR analysis of RNA from (D) at Day 4 post-electroporation, including (E) positive-strand, (F) negative strand, and (G) the ratio of positive-to-negative (+):(-) strand RNA. All data are representative of three independent replicates and error bars represent the standard deviation of the mean.

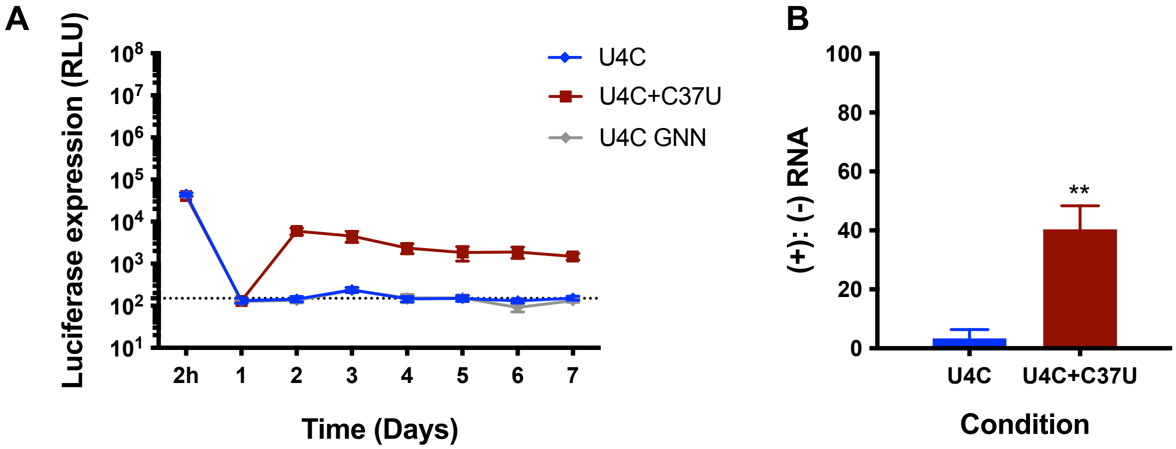

Figure S8. Combining U4C and C37U RAVs rescues viral RNA accumulation in miR-122 KO cells. (A) Full-length Renilla Luciferase (RLuc) HCV genomic reporter RNAs (U4C, U4C+C37U and U4C GNN) were co-electroporated with a capped Firefly Luciferase (FLuc) mRNA into miR-122 KO cells. Luciferase activity was measured at the indicated time points post-electroporation. The limit of detection is indicated. (B) RT-qPCR analysis of U4C and U4C+C37U accumulation in miR-122 KO cells presented as a ratio of positive-to-negative (+):(-) strand RNA. Data are representative of three independent replicates and error bars represent the standard deviation of the mean. Statistical significance was determined by multiple t-test, $**p \leq 0.01$.

Genotype, Accession #	5' terminus (nucleotides 1-63)
	SLI SLII^{alt}
**2a, AB047639(J6,JFH-1)**	-**ACCU**GCCCCUAAUAGGGG-CGA**CACUCC**G**CCA**UGAAU**CACUCC**CCUGUGAGGAACUACUGUCUU
	SLI SLII
1a, AF009606	-G..A.....CUGAU....G.........A...................................
1b, M58335	----------CG..U....G.........A....AG.............................
1c, D14853	-G..A.....CUGAU....G...
2a, D00944	-...C................................C...........................
2b, D10988	-G..C.....CUGAU....G...
2c, D50409	-...C........A...
2k, AB031663	UG..C........AU..
3a, D17763	-.......U..U.CGA..-..........A.....G.....................U.......
3b, D49374	-.......U..UUCGA..-..........A...........................U.......
3k, D63821	-.......U..UUCGA..-..........A...........................U.......
4a, DQ295833*	-------------.U....G.........A....AG.................U...........
5a, D50466*	-...C......UA.U..............A......-............................
6a, D88476*	-G..A......U.AC...................U.-............................
6b, D84262	-G..A......U.AC..............A......-............................
6d, D84263	-C..A.........-..............A......-............................
6f, D63822	-G..A......UAC-..............A......-............................
6h, D84265	-G..A.........-..............A......-............................
6k, D84264	-G..A.........-..............A......-............................

Genotype, Accession #	5’ terminus (nucleotides 64-117)
	SLII^{alt}
**2a, AB047639(J6,JFH-1)**	CACGCAGAAAGCGCCUAGCCAUGGC-GUUAGUAU-GAGUGUCGUACAGCCUCCAGG
	SLII
1a, AF009606	.............U.............................G............
1b, M58335	.............U.............................G............
1c, D14853	.............U.............................G............
2a, D00944	.............U..
2b, D10988	.............U..
2c, D50409	.............U..
2k, AB031663	.............U..
3a, D17763	.....G...........................C.........G............
3b, D49374	.....G.......U...................C.........G............
3k, D63821	.....G.......U...................C.........G............
4a, DQ295833*	.............U...........A........A.....................
5a, D50466*	............U..........................A...........
6a, D88476*	.............U..
6b, D84262	.............U..
6d, D84263	............U......................................
6f, D63822	.............U..
6h, D84265	............U......................................
6k, D84264	.............U..

Figure S9. Comparison of 5’ terminal sequences (nucleotides 1-117) of HCV genotypes 1-6. Representative 5’ terminal sequences (nucleotides 1-117) of HCV clones belonging to each of the confirmed genotypes/subtypes found at euHCVdb: https://euhcvdb.lyon.inserm.fr/euHCVdb/jsp/nomen_tab1.jsp (4). Consensus sequences compared with the JFH-1 isolate (genotype 2a, AB047639) whose 5’ terminus (nucleotides 1-117) is identical to the J6/JFH-1 chimera used herein (Genbank accession: JN180452). The 5’ terminal stem loop (SL) structures, including those in the alternative (SLI and SLII^{alt}) and functional (SLI and SLII) conformations are indicated in orange (top) and blue (bottom), as well as the miR-122 seed and auxiliary sequences (bold), are indicated. The confirmed sequences for genotypes 4a, 5a and 6a are truncated at their 5’ termini, and hence provisionally assigned sequences DQ295833*, D88473*, and D50466* were retrieved for analysis.

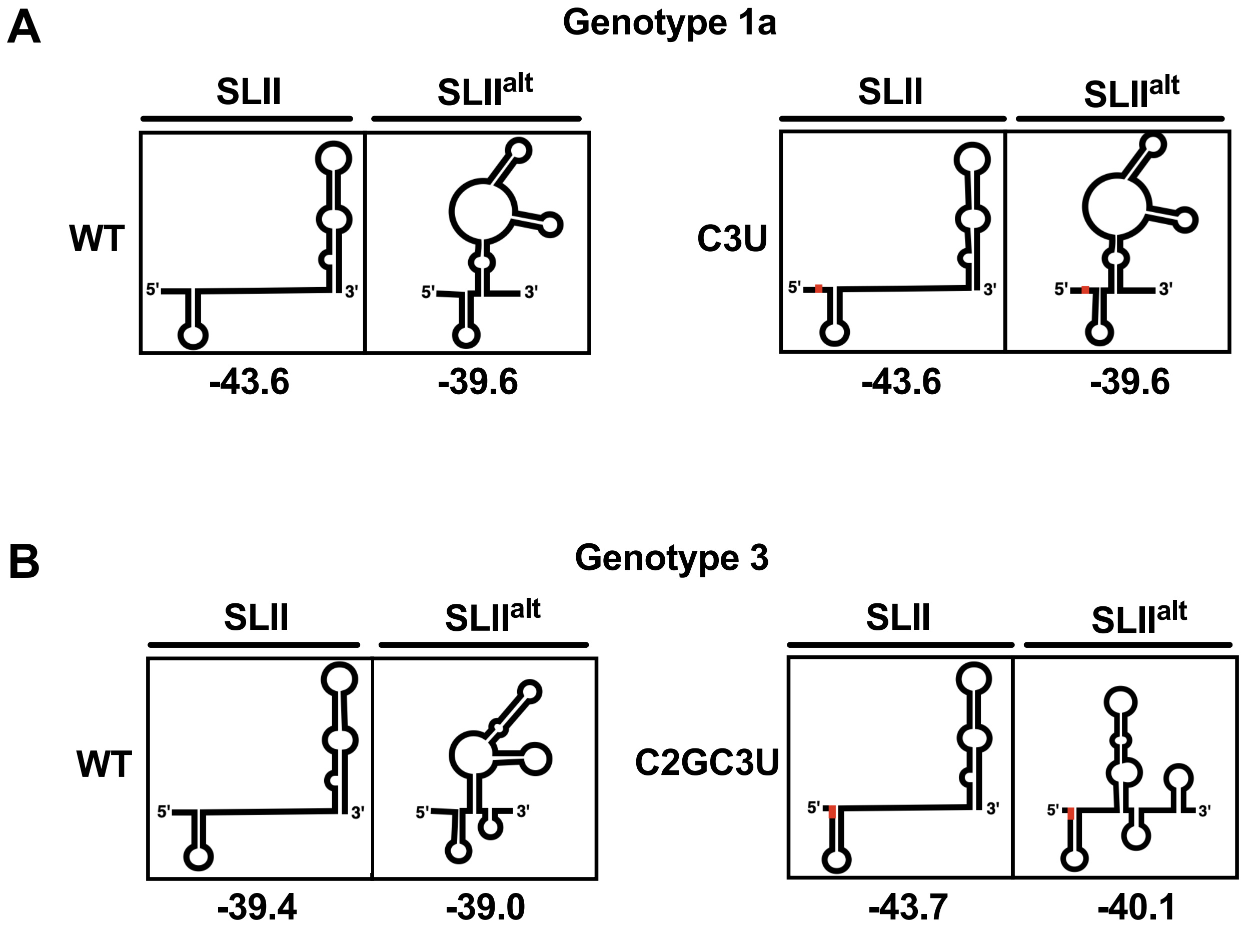

Figure S10. RNA structure predictions of the 3' end (nucleotides 1-117) of the positive-strand HCV genomic RNA. Structural predictions and the predicted free energies (ΔG, kcal/mol) of the functional (SLII) and alternative ($SLII^{alt}$) conformations are indicated. **(A)** Genotype 1a WT and C3U predictions. **(B)** Genotype 3 WT and C2GC3U predictions. The mutations in the HCV RNAs are shown in red.
